# mSphere of Influence: the Wild Genetic Diversity of Our Closest Yeast Companions

**DOI:** 10.1128/mSphere.00650-19

**Published:** 2019-09-25

**Authors:** Douda Bensasson

**Affiliations:** a University of Georgia, Athens, Georgia, USA

**Keywords:** wine yeast, *Candida albicans*, climate

## Abstract

Douda Bensasson uses the population genomics of model yeast species to understand how wild yeast colonize new environments, such as humans or their food. In this mSphere of Influence article, she reflects on how the discovery of “Surprisingly diverged populations of Saccharomyces cerevisiae in natural environments remote from human activity” (Q.-M. Wang, W.-Q. Liu, G. Liti, S.-A. Wang, and F.-Y. Bai, Mol Ecol 21:5404–5417, 2012, https://doi.org/10.1111/j.1365-294X.2012.05732.x) showed that a field survey and population genetic analysis of old growth forests could “unveil the hidden part of the iceberg” of natural variation in S. cerevisiae that went unnoticed for over a hundred years of yeast research.

## Abstract (precis)

Douda Bensasson uses the population genomics of model yeast species to understand how wild yeast colonize new environments, such as humans or their food. In this mSphere of Influence article, she reflects on how the discovery of “Surprisingly diverged populations of Saccharomyces cerevisiae in natural environments remote from human activity” (Q.-M. Wang, W.-Q. Liu, G. Liti, S.-A. Wang, and F.-Y.

## COMMENTARY

Wang et al. (1) searched the primeval forests of China for the yeast species that humans used to make food and drink for millennia and discovered “Surprisingly diverged populations of Saccharomyces cerevisiae in natural environments remote from human activity.” This came at a time when we knew that S. cerevisiae lived wild on the bark of oak trees (2, 3), but there was a world shortage of environmental isolates. Wang et al. (1) showed that the genetic diversity of forest S. cerevisiae isolates on a single Chinese island smaller than Belgium exceeded the genetic diversity seen across all other continents or habitats. This enormous diversity implied that old-growth trees represent the ancestral habitat of S. cerevisiae and finally dispelled the concern that it is too domesticated to be useful as a model for ecology and evolution.

The tropics have always been poorly sampled for yeast (4, 5), and Wang et al. closed this gap by comparing S. cerevisiae isolation frequencies among 11 provinces in tropical, subtropical, and temperate regions (1). By coupling fieldwork with population genetics, they noticed that genetic diversity centered in hot climates, while known domesticated lineages clustered around Chinese cities in temperate regions and could be feral. They therefore proposed that S. cerevisiae is tropical and subtropical. Leaving no loose ends, they also generated mutants for crossing experiments and showed that the new lineages are S. cerevisiae and not new species. Seven years on, the known genetic diversity of S. cerevisiae in Far East Asia has grown and remains unsurpassed despite the discovery of large-scale diversity in Brazilian rainforests (6). The East Asian origin for S. cerevisiae that Wang et al. proposed is probably true (7).

If correct, Wang et al.’s proposal that S. cerevisiae is tropical and subtropical would explain why generations of yeast biologists living in temperate regions believed that S. cerevisiae was a domesticated species with no natural conspecifics. It also would explain why yeast labs in cool temperate climates found few wild S. cerevisiae isolates in their large field surveys (8–11). When Wang et al. published, I realized that my lab had sampled woodlands in the wrong climate. Most immediately, this paper inspired me to develop climate envelope models using field data on the closest relative of S. cerevisiae. The labs in temperate climates (including mine) had no trouble isolating the sister species, Saccharomyces paradoxus (8–11), which prefers cooler temperatures in the lab (12). We used our European S. paradoxus data and the known species difference in thermal preference to show that S. cerevisiae is indeed subtropical and tropical and to correctly predict the temperate locations where only feral S. cerevisiae strains occur in China (11). Unsurprisingly, the interdisciplinary work of Wang et al. influenced me in other ways. It inspired me to combine population genomic analyses with ecological data to show that old oak trees provide a natural habitat for Candida albicans, previously considered an obligate commensal (13). There was also a sociological lesson: despite over a hundred years of research into “man’s best (micro) friend” (14), most of the natural diversity of the model S. cerevisiae remained unnoticed until Wang et al.’s exciting discovery.
